# Spinal muscular atrophy: From approved therapies to future therapeutic targets for personalized medicine

**DOI:** 10.1016/j.xcrm.2021.100346

**Published:** 2021-07-21

**Authors:** Helena Chaytow, Kiterie M.E. Faller, Yu-Ting Huang, Thomas H. Gillingwater

**Affiliations:** 1 Euan MacDonald Centre for Motor Neurone Disease Research, University of Edinburgh, Edinburgh, UK; 2 Edinburgh Medical School: Biomedical Sciences, University of Edinburgh, Edinburgh, UK; 3 Royal (Dick) School of Veterinary Studies, University of Edinburgh, UK

**Keywords:** apoptosis, cytoskeleton, gene therapy, neuroprotection, neuromuscular junction, SMN, splicing modulator, ubiquitination

## Abstract

Spinal muscular atrophy (SMA) is a devastating childhood motor neuron disease that, in the most severe cases and when left untreated, leads to death within the first two years of life. Recent therapeutic advances have given hope to families and patients by compensating for the deficiency in survival motor neuron (SMN) protein via gene therapy or other genetic manipulation. However, it is now apparent that none of these therapies will cure SMA alone. In this review, we discuss the three currently licensed therapies for SMA, briefly highlighting their respective advantages and disadvantages, before considering alternative approaches to increasing SMN protein levels. We then explore recent preclinical research that is identifying and targeting dysregulated pathways secondary to, or independent of, SMN deficiency that may provide adjunctive opportunities for SMA. These additional therapies are likely to be key for the development of treatments that are effective across the lifespan of SMA patients.

## Highlights

Three licensed SMA therapies increase SMN levels, but are not a cure
Other strategies to increase SMN levels are still under development
Alternatives target the correction of dysregulated pathways following SMN loss
Ultimately, a range of therapies may allow for a tailored treatment

## Graphical abstract

**Figure:**
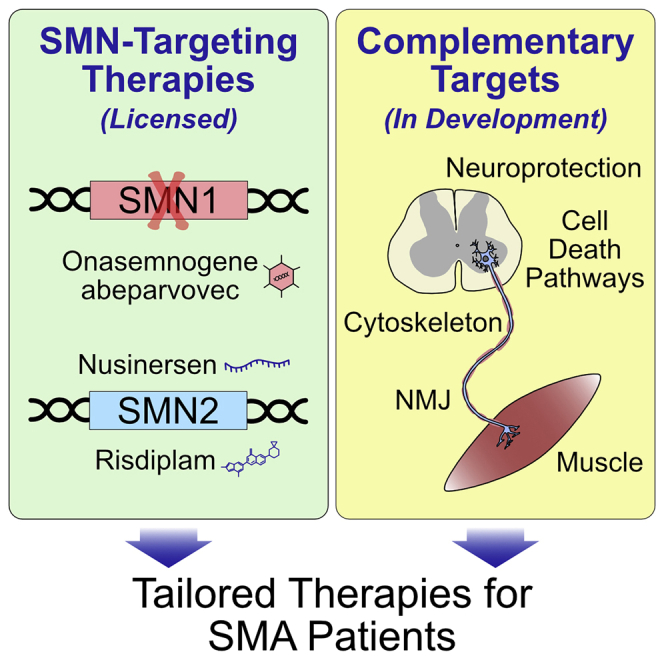

## Teaser

Spinal muscular atrophy, a childhood neurodegenerative disorder, is caused by survival motor neuron (SMN) protein loss. Chaytow et al. review the newly licensed therapies and their limitations, before discussing alternative targets to correct the numerous consequences of SMN loss. Ultimately, a range of therapies may become available, allowing for tailored treatment.

## Introduction

Spinal muscular atrophy (SMA), a childhood-onset motor neuron disease, has historically been the most frequent genetic cause of infant mortality,^1^ although this is likely to change with the recent therapeutic “revolution.” SMA, caused by mutations in the *Survival Motor Neuron 1* (*SMN1*) gene, leads to loss of SMN protein expression. This is partially compensated for by expression from the paralogous gene *SMN2*, which differs from *SMN1* by a single-nucleotide polymorphism in exon 7.^2^^,^^3^ This base change causes exclusion of exon 7 in nearly 90% of *SMN2* transcripts, and the truncated unstable protein (SMNΔ7) is quickly degraded.^4^ The SMN protein is ubiquitously expressed and plays a fundamental role in cell homeostasis through multiple functions, which are still not fully understood.^5^ It is involved in various cell mechanisms, such as the assembly of the spliceosomal machinery, endocytosis, and protein translation. Because of its diverse roles and ubiquitous expression, loss of SMN can lead to systemic pathology extending beyond the motor neuron, which has to be considered when designing new therapies.^6^

Within SMA, disease severity varies, at least in part, according to the number of *SMN2* copies carried by the patient.^7^ In SMA type 0/1, the most frequent form with one copy of *SMN2*, untreated children will never have the muscular strength to sit unassisted, with a life expectancy of around 2 years. As patients carry more *SMN2* copies, the severity of the disease decreases up to type 4, characterized by an early-adulthood clinical onset and typically a normal life expectancy.^1^ However, this natural history of the disease will likely become obsolete as the majority of patients will receive at least one disease-modifying therapy.

As a rare example of a monogenetic neurodegenerative disease, SMA research has pioneered gene-targeted therapy with the recent approval of three therapies designed to enhance SMN production. Regardless of this transformation in SMA therapy, it has become apparent that these novel treatments are still not a cure. In this review, we first describe the three approved therapies before detailing alternative strategies aimed at increasing SMN levels. Finally, we discuss other potential targets for drug development.

## Gene-targeting SMN replacement therapies

Currently, three treatments—nusinersen (Spinraza; Biogen), onasemnogene abeparvovec (Zolgensma; Novartis), and risdiplam (Evrysdi; Roche)—are approved by the US Food and Drug Administration (FDA) and the European Medicines Agency (EMA) (Table 1). Here, we discuss the development of these three drugs in chronological order of licensing as well as ongoing efforts to improve these strategies.

**Table 1:** Summary of selected SMN-targeted therapies approved or in clinical development

Drug/company	Mechanism of action	Stage of development	Route of administration and protocol	Population targeted by the license	Cost^8^^a^	Other comments
**Approved therapy**						

Nusinersen (Spinraza)/Biogen	splicing modifier of *SMN2* (antisense oligonucleotide)	approved by the FDA (December 2016) and the EMA (May 2017)	intrathecal administration: 3 loading doses at 14-day interval, 4th loading dose 30 days after the 3rd dose, and maintenance dose every 4 months thereafter	all ages and all types of SMA	up to $125,000 per dose; drug cost for the first year: $750,000 and then $375,000 annually; rebates have been obtained by some countries and organizations, but in most cases, this is not transparent	long-term efficacy and side effects unclear
						intrathecal administration difficult/impossible for patients who had surgeries for scoliosis, making nusinersen not an option for these patients
						optimum dosing and protocol have been underexplored, and ongoing trials are evaluating the potential of higher dosing (NCT04089566)
						approved for use in adults, despite the lack of clinical trials on adults at the time of approval and unknowns regarding the dose; first studies suggest it could be promising in some patients,^9^^,^^10^ but further trials are required
						targets the central nervous system
Onasemnogene abeparvovec-xioi (Zolgensma)/Novartis	replacement of *SMN1* gene	approved by the FDA (May 2019) and the EMA (conditional approval May 2020)	intravenous injection (single dose)	FDA: treatment of pediatric patients less than 2 years of age with SMA with bi-allelic mutations in the *SMN1* gene. EMA: patients with 5q SMA with a bi-allelic mutation in the *SMN1* gene and a clinical diagnosis of SMA type 1, or patients with 5q SMA with a bi-allelic mutation in the *SMN1* gene and up to 3 copies of the *SMN2* gene	$2,125,000 (single injection)	limited experience in patients over 2 years old
						requires an immunomodulatory^11^ regimen with prednisolone before and after intravenous infusion (for at least 2 months including tapering period) to decrease the response to the AAV9 capsid
						long-term efficacy and safety unclear
						thought to remain primarily in post-mitotic cells (e.g., neurons); hence, a not true systemic effect
						irreversible treatment
Risdiplam (Evrysdi)/Roche	splicing modifier of *SMN2* (small molecule)	approved by the FDA (August 2020)^12^ and the EMA (March 2021)^13^	oral, once daily	patients 2 months of age and older	up to $340,000 a year (cheaper in younger patient as dosing is weight related)	long-term efficacity and safety unclear
						oral administration allows for systemic treatment
**In clinical development**						

Branaplam/Novartis	splicing modifier of *SMN2* (small molecule)	still under development; enrollment in phases I and II completed, but results have not yet been released^14^	oral; still under development (weekly administration in the clinical trial)	not yet applicable	not yet applicable	initial enrolment had been halted in 2016 due to safety concerns
				current clinical trial focused on type 1 with 2 *SMN2* copy numbers		resumed in end 2017 after amendments to protocol (NCT02268552)
						oral administration would allow for systemic treatment

a The costs given are indicative and do not include hospitalization and procedure fees.

### Nusinersen

Since manipulation of the splicing pattern of the *SMN2* gene can produce full-length, functional SMN protein, strategies that target splicing regulatory elements and boost exon 7 inclusion have shown great success and were the first SMN replacement therapy to achieve regulatory approval. Exclusion of exon 7 from the *SMN2* transcript is regulated by a section of intron 7 termed the intronic splicing silencer N1 (ISS-N1), located immediately downstream of the 5′ splice site for exon 7, and deletion of the ISS-N1 sequence significantly increased exon 7 inclusion.^15^ Manipulation of such regulatory regions by short complementary sequences of synthetic nucleotides is an important therapeutic approach. To determine the most effective target sequence for exon 7 inclusion, multiple antisense oligonucleotides (ASOs) were tested in mice with the sequence shifting along the target by one base at a time.^16^ The strongest sequence (intron 7 position +10-27) was injected as an 18-mer 2′-O-methoxyethyl-modified ASO into a mild mouse model at embryonic, neonatal, and adult stages. SMN protein levels increased, corresponding to an improvement of the SMA phenotype, and early treatment (embryonic or neonatal) gave a stronger phenotypic correction.^17^ The specific ASO-10-27 was able to dramatically improve lifespan, motor function, and muscle physiology by either systemic or intracerebroventricular injection in two severe SMA mouse models.^18^^,^^19^ Preclinical studies in mouse models and non-human primates showed good distribution of the ASO throughout the spinal cord and dose-dependent effects on SMN expression following a single intrathecal infusion.^19^^,^^20^ Central nervous system (CNS) and systemic delivery of ASOs have to be considered as complementary strategies, since a combined systemic and CNS-directed delivery strategy had the strongest effect on both survival and vascular-related clinical signs in a severe mouse model of SMA.^21^

Based on this preclinical evidence, clinical trials were initiated and subjects were found to safely tolerate multiple intrathecal injections in phase II, with evidence of improvement of motor function and achievement of motor milestones.^22^ Successful phase III data led to FDA and EMA approval in December 2016 and June 2017, respectively, as the first drug to treat 5q SMA type 1–3 patients (Table 1). Infants treated before 6 months or later, in ENDEAR and CHERISH trials, respectively, showed positive results in terms of motor milestones and event-free survival; hence, both phase III trials were terminated at the interim stage to allow all participants to move onto nusinersen in an open-label study (SHINE).^23^

The most recent data from clinical trials for nusinersen provide evidence of long-term safety and efficacy across patient groups, with dramatically improved survival and motor function.^23^^,^^24^ After approximately three years of treatment, 100% of SMA type 2 patients were able to sit unsupported, with some able to walk with support, while 76% of SMA type 3 patients could walk independently.^24^ Results also suggest that early treatment may maximize efficacy. Those in the NURTURE clinical trial, which treated infants pre-symptomatically before 6 weeks of age for the first dose, had higher motor function at every point of observation after treatment, with the latest data showing 22 of 25 children able to walk independently and none requiring permanent ventilation.^25^ Nusinersen is now available in many countries, with a wider perspective on its efficacy in a range of patient groups,^26^, ^27^, ^28^ and some evidence that clinical improvement continues after the first year of treatment.^29^ Finally, a recent study assessed the distribution of ASO and the associated SMN increase in the spinal cord of post-mortem samples from infants treated with nusinersen. This study suggests that there is variability in distribution of ASO within the CNS, with a lower concentration in the cranial portion of the spinal cord and in the brain.^30^ However, these results need to be put in perspective, considering these patients died in infancy and as such were poor responders to the therapy.

### Onasemnogene abeparvovec

Since the causative factor of SMA is a monogenic defect leading to the loss of SMN protein, this motor neuron disease is a prime candidate for gene replacement therapy. The idea of using viral vectors to deliver exogenous genes to relevant tissues was expanded to neurodegenerative diseases when it was demonstrated that systemic delivery via intravenous (i.v.) injection of adeno-associated viral vector serotype 9 (AAV9)-based gene transfer can cross the blood-brain barrier (BBB)^31^ and efficiently transduce target cells in the CNS, including motor neurons in the spinal cord in mice and non-human primates.^31^^,^^32^ Development of the self-complementary AAV9 (scAAV9) vector further improved the efficiency and speed of gene transcription.^33^ Using this technique, AAV9-mediated SMN gene expression delivered at postnatal day 1 (P1) significantly improved lifespan and motor symptoms in SMA models.^32^, ^33^, ^34^ Initial studies also showed that early treatment of SMA mice at P1 generated better outcomes compared with treatment at P5 or P10, highlighting the importance of early intervention in SMA therapy.^34^

These successes led to the initiation of a phase I clinical trial of onasemnogene abeparvovec (previously AVXS-101; onasemnogene hereafter): a recombinant AAV9 viral vector encoding human SMN protein under the control of the cytomegalovirus enhancer/chicken-β-actin hybrid promoter injected i.v. This first trial treated 15 infants, 3 with low dose and 12 with high dose: all 15 patients survived to 20 months without the need for respiratory support, with 11 patients reaching the motor milestone of sitting unassisted and 2 even walking independently.^35^ This positive phase I trial and interim data released from the successive phase II/III trial led to FDA approval in 2019 (Table 1). However, systemic administration by i.v. of high-dose AAV9-SMN was found to cause liver dysfunction in non-human primates.^36^ This has led to the temporary suspension of an ongoing clinical trial (NCT03381729) intending to administer high-dose onasemnogene intrathecally, pending further clarification on preclinical findings. Nevertheless, in a phase III clinical trial, 22 patients with symptomatic type 1 SMA received onasemnogene; of these, 13 infants achieved independent sitting at 18 months of age. Ninety-one percent of patients did not require permanent ventilation by the age of 14 months, compared with only 26% of the untreated group.^37^ Other trials include phase III STR1VE-EU (NCT03461289) and STR1VE-AP (NCT03837184), which have been assessing efficacy and safety in SMA infants under 6 months of age with 1 or 2 copies of *SMN2*, and SPR1NT (NCT03505099), which enrolled SMA infants under 6 weeks old to evaluate the efficacy and safety in pre-symptomatic patients. Liver toxicity remains so far the main adverse event, with the majority of patients receiving onasemnogene also requiring prednisolone treatment to mitigate the hepatotoxicity.^11^

### Risdiplam

Risdiplam (RG7916), a small-molecule splice modulator, was approved by the FDA in 2020 and the EMA in 2021. Risdiplam acts as an *SMN2* splice modulator, directly binding to *SMN2* pre-mRNA at two sites, an exon enhancer sequence and the 5′ splicing site of exon 7, stabilizing the ribonucleoprotein complex and competing with hnRNPG binding, thus promoting exon 7 inclusion and full-length SMN protein production.^38^ Risdiplam was optimized from RG7800, a splice modulator found to increase full-length *SMN2* mRNA 2-fold in healthy adults and SMA patients.^39^ However, development of RG7800 was put on hold due to retinal damage in non-human primates.^40^ Risdiplam belongs to the SMN-C class of splice modulators: it increased full-length SMN protein in both severe and mild mouse models of SMA, with an increase in survival and improvement of motor phenotypes.^41^

Oral dosing of risdiplam at 3 mg/kg/day for 7 days in non-human primates showed good biodistribution in relevant tissues,^42^ while a phase I trial confirmed good tolerance and no evidence of retinal pathology.^43^ Recently published results from the phase II/III FIREFISH study determined improved efficacy at the higher tested dose, for which 7 of 17 infants were able to sit independently after 12 months of treatment. A follow-up study is ongoing, focusing exclusively on the long-term effects of the higher dose.^44^ Further phase III clinical trials are ongoing, with SUNFISH assessing effectiveness in type 2/3 patients aged 2 to 25 years old (NCT02908685), JEWELFISH investigating the effects of risdiplam in patients previously treated with other SMA therapies (NCT03032172), and RAINBOWFISH studying risdiplam in pre-symptomatic SMA infants under 6 weeks old at first dose (NCT03779334). Extensive ophthalmologic assessment has also been performed in the patients enrolled in the FIREFISH, SUNFISH, and JEWELFISH clinical trials and did not reveal any retinal toxicity.^45^

### Other potential drugs targeting SMN2 splicing

Methods of increasing SMN expression via splice modulation continue to be researched for improved efficacy. Branaplam was identified using a high-throughput screen for *SMN2* exon 7 inclusion and appears to stabilize *SMN2* pre-mRNA with splicing factor complexes.^46^ Daily administration showed a dose-dependent increase of exon 7 inclusion and SMN protein expression in SMA mice, with an improvement in body weight and lifespan.^46^ The first in-human phase I/II trial started in 2015 across trial centers in Europe (NCT02268552) treating SMA patients younger than 6 months old with 2 copies of *SMN2* (Table 1). Interestingly, Novartis has announced a finding of reduced huntingtin mRNA (the mutated protein in Huntington’s disease) in SMA patients treated with branaplam, which has formed the basis of the FDA’s Orphan Drug Designation for branaplam in Huntington’s disease, with a phase IIb trial planned in 2021.^47^

*In vitro* splicing screens on *SMN2* have identified several novel small molecules that modulate splicing patterns and improve exon 7 inclusion, including the identification of risdiplam and branaplam. Additional molecules from the screen initially identifying risdiplam continue to be developed to further enhance *SMN* splicing.^48^ Another recently discovered small molecule, TEC-1, with a similar chemical structure to risdiplam, has demonstrated increased SMN expression, increased lifespan, and improved motor phenotypes in a severe SMA mouse model, with fewer off-target splicing changes than risdiplam.^49^ Further screens have identified that flunarizine, a calcium channel blocker, can alter splicing events in HeLa cells, including intron retention in *SMN.*^48^ This was confirmed in a screen on SMA patient fibroblasts, where flunarizine increased localization of SMN to Cajal bodies and improved the survival of spinal cord motor neurons from SMA mice.^50^

Alternatively, while nusinersen shows a strong effect on splicing and good biodistribution and pharmacokinetics, it may be possible to improve these outcomes through adjusting the ASO’s target or chemistry. As such, optimization of ASOs targeting *SMN2* splicing continues, with alternative potential target sequences^51^ and improved chemistries, including cell-penetrating peptides.^52^

### Critical appraisal of current therapies

These three licensed therapies have been at the forefront of the therapeutic “revolution” for SMA. However, there remain some major limitations and concerns. First, nusinersen must be delivered via invasive intrathecal injection multiple times per year. This leads to CNS-specific distribution, meaning that systemic symptoms may not be fully addressed. Onasemnogene is administered i.v., so it will have systemic distribution, but there remains the issue of bioavailability of the AAV9 serotype and the fact that long-term transgene expression is expected to be limited to post-mitotic cells such as neurons. Conversely, risdiplam is a systemic therapy and is the least invasive licensed treatment with daily oral administration. However, as risdiplam targets the splicing machinery, it may also affect other transcripts, leading to unknown off-target side effects. For example, although risdiplam appears to have very high specificity to *SMN2*, the cell division regulator *FOXM1* is also targeted by risdiplam at high concentrations, risking oncogenic side effects,^40^ so dosage has been strictly monitored in clinical trials. The long-term effects of all three licensed therapies are currently unknown and may only become apparent many years after onset of treatment. As it stands, through necessity, most available clinical data are through clinical trials funded by pharmaceutical industries, with specific inclusion criteria and short follow-up. Real-world data from a wide spectrum of patients over an extended period of time are slowly becoming available and highlight the shortcomings of current SMN replacement therapies. Treated children remain disabled with complex needs and high level of care requirements.^26^^,^^27^^,^^53^ Moreover, while none of these therapies represents a cure for SMA, each of these therapies carries a high price tag. The one-off onasemnogene injection is priced at $2.1 million, the most expensive drug in the world, with nusinersen costing $750,000 in the first year followed by $375,000 annually thereafter, and risdiplam priced at up to $340,000 per year.^8^ These costs can clearly lead to major issues for patients and their families as well as healthcare providers (Table 1). Furthermore, although onasemnogene is advertised as a single-injection therapy, it remains unclear whether the treatment will be lifelong or whether additional therapies will be required. Currently, no clinical guidelines are available to help clinicians and families choose one therapy over another for a specific patient. Direct comparison of these therapies is made difficult due to different inclusion criteria, assessment, and outcome measures across clinical trials. This is a critical issue that will have to be addressed through unbiased clinical trials specifically designed for comparison.

## Wider therapeutic strategies: Beyond the gene-targeting drugs

As mentioned above, these currently licensed therapies have changed the phenotype of SMA patients. However, the clinical trials and real-world data highlight the need for adjunctive therapies in order to improve the quality of life of these patients. Research has therefore focused on addressing the systemic consequences of SMN loss. As such, the potential targets are not specific to SMA, but rather fit in a wider strategy for treatment of neuromuscular disorders. A drug that is already approved or in clinical trials for one condition may also be advantageous in SMA, in combination with SMN replacement therapies or alone. This repurposing approach could result in a faster path to the patient due to already available safety profiles. In the following section, we discuss these broader strategies and highlight those that are already in the clinic for various conditions in Table 2.

**Table 2:** Summary of drugs with repurposing potential in SMA

Name of molecule	Mechanism of action for treatment of SMA	Licensed (indication)	Clinical trials in SMA				Clinical trials in other neurological/neuromuscular diseases
			Trial number	Phase	Patient group	Results	
**Non-specific therapies increasing SMN levels**							

Flunarizine	splice modulation	Y (not in all countries - migraines)	NA				NA
Valproic acid	HDAC inhibitor - increased SMN expression	Y (bipolar disorder, migraine prophylaxis, epilepsy)	meta-analysis of clinical trials up to 2017 indicated an improvement in motor function, but not survival^54^				ALS - NCT00136110 (phase III): completed, no results posted, NCT03204500 (phase II): completed, no results posted
Sodium phenylbutyrate	HDAC inhibitor - increased SMN expression	Y (urea-cycling disorders)	NCT00528268	I/II	Presymp. type 1/2	completed 2015	ALS - NCT03127514 (phase II): part of combination treatment PB-TURSO, results showing improvement in motor function and survival^55^ IBM - NCT04421677 (phase I): ongoing
				I/II		secondary outcomes of improved motor function and body mass not reported	
			NCT00439569	I/II	type 2/3	terminated due to poor compliance	
			NCT00439218		type 1	terminated due to slow recruitment	
SAHA (vorinostat)	HDAC inhibitor - increased SMN expression	Y (lymphoma)	NA				AD - NCT03056495 (phase I): recruiting
Resveratrol	HDAC inhibitor - increased SMN expression	Y (dietary supplement)	NA				ALS - NCT04654689 (phase II): not yet recruiting
							muscular dystrophies (phase IIa): improvement in muscle function^56^
LBH589 (panobinostat)	HDAC inhibitor - increased SMN expression	Y (myeloma)	NA				NA
Azithromycin	increased SMN expression	Y (antibiotic)	NA				NA
Bortezomib	increased SMN expression	Y (myeloma/lymphoma)	NA				MG - NCT02102594 (phase II): terminated due to low recruitment

**Neuroprotection**							

Terazosin	PGK1 activation	Y (hypertension)	NA				NA
Olesoxime	mitochondrial protection	N	2006-006845-14	Ib	type 2/3	well tolerated	ALS - NCT00868166; NCT01285583 (phase II/III): add-on to riluzole, with no effect on survival or motor function MS - NCT01808885 (phase I): no results posted
			NCT01302600	II	type 2/3	well tolerated, no change in motor function	
			NCT02628743	II	type 2/3	long-term motor decline, matched to natural history control data	
Riluzole	glutamate receptor antagonist	Y (ALS)	NCT00774423	II/III	type 2/3	no results posted	ALS - Cochrane systematic review based on 4 clinical trials suggests that riluzole increases life expectancy by 2–3 months^57^
Gabapentin	VGCC inhibitor	Y (focal seizures and others including muscular symptoms in ALS)	–	II/III	type 2/3	no effect on any outcome measure^58^	NA
			–	II/III	type 2/3	improvement in limb strength tests, but no change in respiratory tests^59^	
Edaravone	antioxidant	Y (ALS - US and Japan only)	NA				ALS - NCT00330681
							(phase III): no significant functional difference; nevertheless, post hoc analysis suggested it could be effective in patients with shorter disease duration and milder symptoms
							NCT01492686 (phase III): restricted to patients with shorter disease duration and milder symptoms: slower functional decline in treated patients
Levetiracetam	anti-epileptic	Y (epilepsy)	NA				ALS - NCT00324454 (phase II): no results posted
							AD - NCT03489044 (phase II): no results posted

**Muscle targeting**							

ACE-031	ActRII inhibitor	N	NA				DMD - NCT01099761 (phase II): trend toward improved muscular function and increased lean body mass; study discontinued due to side effects (telangiectasia and epistaxis)
Bimagrumab	ActRII inhibitor	N	NA				sporadic IBM - NCT01925209 (phase IIb): no functional improvement
							NCT02573467 (phase III): long-term extension of same study (2 years) did not show any functional benefit
							sarcopenia - NCT02333331 (phase II): no significant functional benefit
Domagrozumab	myostatin inhibitor	N	NA				DMD - NCT02310763 and NCT02907619 (phase II): no significant functional improvement
BIIB110	ActRIIA/B ligand trap	N	–	I	–	no results posted^60^	
Apitegromab (SRK-015)	selective myostatin inhibitor	N	NCT03921528	II	type 2/3	preliminary results indicate improved HFMSE score	
Tirasemtiv (CK-2017357)	FSTAs	N	NA				ALS - NCT02496767 (phase III): no significant difference in the primary outcome measure (SVC) or any secondary outcome measures; poor tolerability
Reldesemtiv (CK-2127107)	FSTAs	N	NCT02644668	II	types 2/3/4	improved maximum expiratory pressure in the highest dose group; post hoc analysis also showed a significant positive change in the 6MWD at 4 weeks, but this was not significant at 8 weeks (p = 0.058)^61^	NA
Somatotropin (somatropin; GH)	anabolic effect	Y (GH deficiency)	NCT00533221	II	type 2/3	no significant effect on muscle strength and function	NA
Recombinant IGF-1 (mecasermin)	anabolic effect	Y (growth failure)	NA				ALS - Cochrane systematic review showed a slight but significant difference in AALSRS total score (based on 2 clinical trials); the third study included in the meta-analysis did not show any significant difference in muscle strength; the quality of all three clinical trials was low^62^ DMD - NCT01207908 (phase II): increase in lean mass, but no significant difference in muscle function
BVS857	IGF-1 mimetic	N	NA				SBMA - NCT02024932 (phase II): significant difference in thigh muscle volume, but no difference in muscle strength and function
Leuprorelin	gonadotropin releasing hormone (GnRH) analogue	N	NA				SBMA - UMIN000000474 (phase II): significant delay in functional decline and a decrease in the incidence of pneumonia and death

**Neuromuscular junction**							

Pyridostigmine	AChE inhibitor	Y (MG)	NCT02941328	II	types 2/3/4	trial completed, but final results not yet published; preliminary reports show a reduction in fatigability^63^	NA
Salbutamol	β2-adrenoreceptor agonists	Y (asthma)	no large-scale clinical trials				MG - NCT03914638 (phase II/III): recruiting
			small clinical studies or case reports in SMA types 2 and 3 suggest a benefit on motor^64^ and respiratory function^65^				FSHD - NCT00027391: results not posted; previous trial did not show any improvement in muscle function^66^
4-Aminopyridine (4-AP)	blocking K^+^ channels	Y (MS)	NCT01645787	II/III	type 3	no improvement on motor function (6MWT distance, fatigue)	PLS - NCT02868567 (phase I): active
Amifampridine (3,4-DAP)	blocking K^+^ channels	Y (Lambert-Eaton myasthenic syndrome)	NCT03781479	II	type 3	no results posted	MG - NCT03579966 (phase III): active

**Cytoskeleton**							

Fasudil	ROCK inhibition	Y (limited countries only - prevention and treatment of cerebral vasospasm)	NA				ALS - NCT03792490 (phase II): currently recruiting

**Cell death pathways**							

MW150	p38α MAPK inhibitor	N	NA				AD - (phase I): ongoing
Celecoxib	NSAID (pain and inflammation)	Y	NCT02876094	II	type 2/3	study terminated; no results posted	ALS - NCT04165850 (phase II)/ NCT00355576 (phase II): results not posted

These drugs are either licensed or currently in clinical trials for other indications, but also have a therapeutic effect in preclinical SMA models. With known safety profiles, these therapies could be “repurposed” for SMA and thus have a potentially faster route to the clinic. Information is accurate as of March 2021.

6MWT, 6 minute walk test; AChE, acetylcholinesterase; AD, Alzheimer’s disease; FSTA, fast skeletal muscle troponin activator; GH, growth hormone; IBM, inclusion body myositis; MAPK, mitogen-activated protein kinase; MG, myasthenia gravis; MS, multiple sclerosis; PLS, primary lateral sclerosis; presymp., pre-symptomatic; SBMA, spinal-bulbar muscular atrophy; SVC, slow vital capacity; VGCC, voltage-gated calcium channel; Y, yes; N, no; NA, not applicable.

### Non-specific therapies increasing SMN levels

Prior to the development of gene-targeting SMN replacement therapies, multiple approaches have been sought to increase SMN levels using drugs not specifically targeting the SMN gene. Although in the current landscape these drugs may seem redundant, they may be considered as an additional therapy to further enhance SMN expression.

#### HDAC inhibitors

Histone deacetylase (HDAC) inhibitors have been investigated in SMA models since the early discovery that histone acetylation controls SMN expression.^67^, ^68^, ^69^ Multiple HDAC inhibitors are either currently licensed or in clinical trials for cancer treatments (Table 2); hence, they make attractive therapies for alternative indications due to their known safety profiles. Screening HDAC inhibitors in SMA patient-derived neuronal cells showed that targeting class I HDACs in particular could boost SMN expression.^70^

Valproic acid is a classic class I HDAC inhibitor that has shown beneficial effects in mouse models of SMA^71^ and patient fibroblasts,^68^ so it was quickly moved in to clinical trials. A systematic review and meta-analysis of valproic acid clinical trials up to 2017 suggested an overall beneficial effect in motor function, but little evidence of change in survival.^54^ Another class I HDAC inhibitor, phenylbutyrate, showed promising levels of SMN expression elevation in patient fibroblasts,^69^ but showed extremely variable outcomes in patients,^72^ and the clinical trial was prematurely terminated (NCT00439569). Other small molecules with HDAC inhibitor properties include suberoylanilide hydroxamic acid (SAHA^73^), trichostatin A,^74^ and resveratrol.^75^ Although these molecules showed success in laboratory models of SMA, they have not been progressed to the clinic.

HDAC inhibitors alone cannot provide a therapeutic benefit to the same levels as SMN replacement.^54^ However, they may provide additional neuroprotective support in combination with other SMN-targeting therapies (“SMN+” therapies^76^). This idea is exemplified in a recent paper showing the benefits of combinatorial therapy between the HDAC inhibitor LBH589 (panobinostat) and low doses of Spinraza-like ASOs.^77^

#### R-loops

During transcription, the double-stranded DNA structure is broken to allow new RNA transcripts to be created, and the DNA-RNA structure with the free non-coding DNA strand is called an R-loop. This physiological process is tightly modulated by nuclear factors and DNA/RNA-binding proteins. However, R-loops can be formed, or not resolved properly, under pathological conditions, thereby disrupting physiological processes and leaving the single-stranded DNA more susceptible to degradation. Suggested mechanisms for pathological R-loop formation have been mutations in, or up/downregulation of, factors controlling R-loop generation or particularly G-rich DNA sequences. Since R-loop formation can be governed by splicing factors, and SMN has a key role in assembly of the spliceosomal small nuclear ribonucleoproteins so its loss leads to widespread splicing defects, these two pathologies may be linked. In cell culture, *SMN1* knockdown led to increased numbers of R-loops over retained introns, and overexpression of RNase H1 (a factor that helps resolve R-loops) prevented DNA damage.^78^ Pathological R-loop formation is therefore a potential therapeutic target, but using DNA-binding molecules as therapies comes with an obvious mutagenic risk. Senataxin, on the other hand, is a DNA repair factor that co-localizes with SMN in Cajal bodies and has a decreased expression in SMA models. Overexpression of senataxin in SMA mouse spinal cord motor neurons reduced R-loop formation and DNA damage.^79^ Another nuclear factor, zinc-finger protein ZPR1, was also found to have reduced expression in SMA models.^80^ Its overexpression doubled the survival of an intermediate mouse model, improved their righting reflexes, and increased motor neuron survival and muscle fiber diameter.^80^ ZPR1 overexpression was shown to increase expression of SMN itself, but could clearly have more global protective pathways as well.

#### Stabilizing the SMN protein

Preventing the degradation of SMNΔ7, the product of *SMN2*, thus allowing even low-level expression to have a more pronounced effect on intracellular pathways, could be a therapeutic strategy. Indoprofen is a non-steroidal anti-inflammatory drug (NSAID) that was identified in an *SMN2-*luciferase screen to increase SMN protein levels in patient fibroblasts.^81^ This screen was also used to identify other compounds that increase SMN expression *in vitro* and *in vivo*.^82^ A novel aminoglycoside, TC007, was found to act as a readthrough compound for exon 8 of *SMN2*, increasing the number of nuclear gems in patient fibroblasts,^83^ and it can slightly increase survival of the severe SMA mouse model.^84^ Other readthrough compounds such as azithromycin have shown some efficacy in mouse models.^85^ Finally, preventing degradation of SMN using the proteasome inhibitor bortezomib improved survival and motor outcomes in SMA mouse models.^86^ However, none of these approaches have yet reached clinical trials for SMA (Table 2).

### Neuroprotection

Since motor neurons are the most severely affected cell type in SMA, it follows that neuroprotective strategies targeted at this neuronal population may be effective, in particular when used in combination with SMN-restoring therapies (SMN+ therapies^76^).

#### Bioenergetics

Neurons and muscle, the major tissue types affected in SMA, have particularly high energy demands; thus, targeting energy pathways may be neuroprotective and therapeutic in SMA. The glycolytic enzyme phosphoglycerate kinase 1 (PGK1) was found to be dysregulated in SMA mouse models, and increasing its activity pharmacologically with terazosin or its expression genetically could ameliorate motor axon phenotypes in SMA zebrafish models.^87^ Alternatively, olesoxime, a mitochondria-targeting therapy, was originally shown to promote cell survival under stressed conditions *in vitro*, likely via modulation of mitochondrial membrane permeability.^88^ As such, olesoxime could be broadly applicable across neurodegenerative diseases and was found to triple the lifespan of the SMA severe mouse model.^88^ Olesoxime showed a good safety profile in type 2/3 patients as well as an improvement in motor function (Table 2).^89^ It was therefore moved on to phase III clinical trials, but the trial was cancelled by Roche amidst the progression of nusinersen, onasemnogene, and risdiplam due to reported issues with dosage and production. However, due to the current pressing demand for combinatorial treatments, olesoxime may return as a subject for future research.^90^

#### Excitotoxicity

An early strategy in SMA research was to test efficacious drugs from other neurodegenerative models, particularly amyotrophic lateral sclerosis (ALS) (Table 2); thus, drugs that target excitotoxicity have been tested in SMA. Riluzole was used in a small preliminary phase I trial based on its modest effects in ALS, which gave a suggestion of an effect with a sample size of 7.^91^ A phase II/III trial to evaluate efficacy of riluzole in SMA patients was completed in 2013, but no results have been posted (NCT00774423). Gabapentin is another drug targeting excitotoxicity that was tested in type 2/3 SMA patients based on its effect in ALS, showing some effect on motor function in one study,^59^ but none in another.^58^ Edaravone, a drug approved for ALS in the US and Japan, showed some promise in SMA patient-derived induced pluripotent stem cell (iPSC)-motor neurons,^92^ but has not yet been taken further. The same group found levetiracetam, an anti-epileptic drug, to have therapeutic potential in their *in vitro* SMA model.^93^

### Muscle-targeting therapies

**Figure 1:**
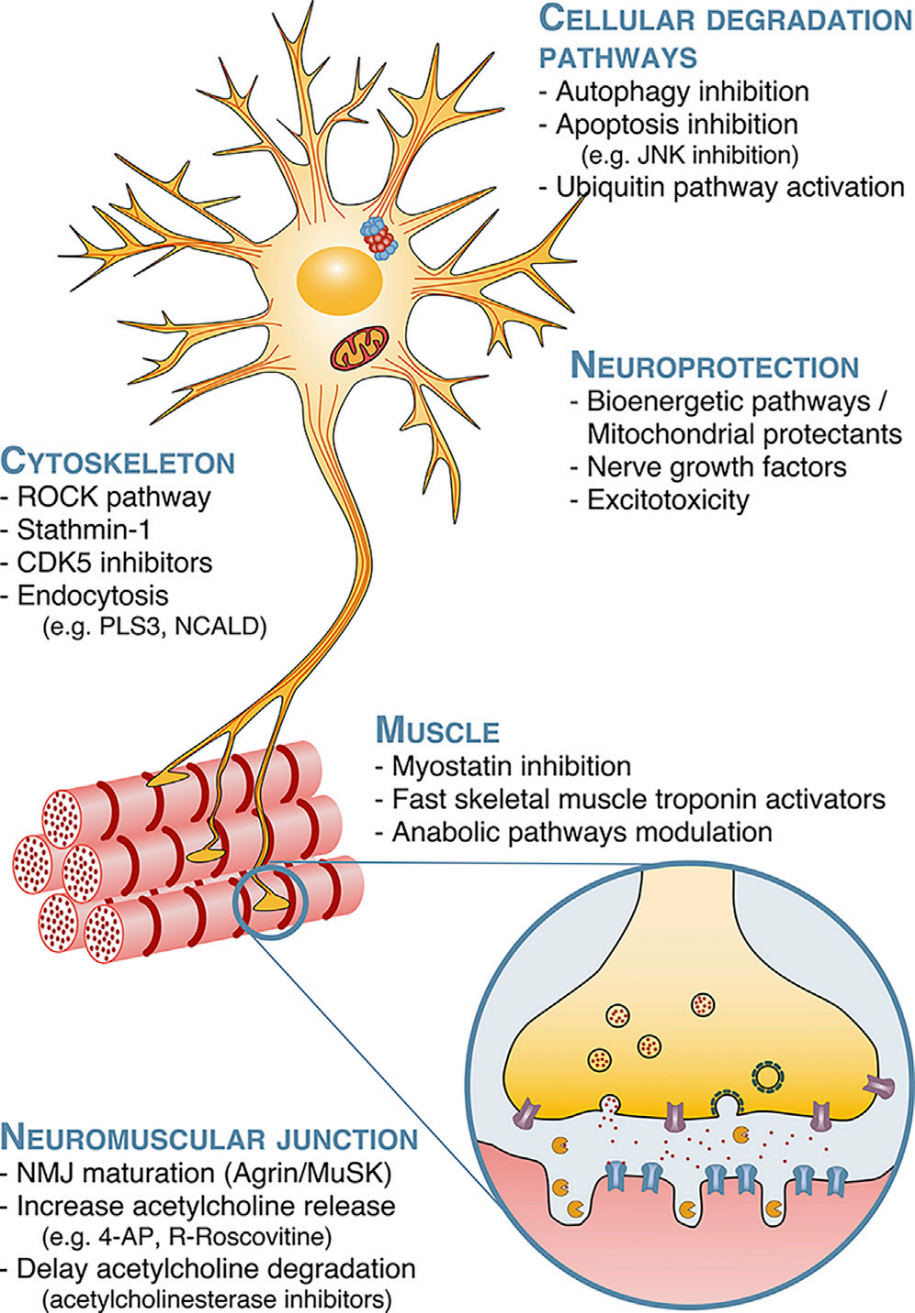
Schematic of the main SMN-independent potential therapeutic targets Because of the diverse cellular roles and ubiquitous expression of SMN, SMN deficiency leads to changes in numerous cellular processes and organs, which have been identified as possible therapeutic targets. For clarity, we classified these targets into cellular pathway degradation, neuroprotection, cytoskeleton, muscle, and neuromuscular junction, but some therapies may span over multiple targets.

Intrinsic and denervation-induced muscle pathology plays an important role in SMA. This was recently confirmed by an elegant study where selective depletion of SMN in skeletal muscle of mice was enough to induce muscular and neuromuscular junction (NMJ) pathology.^94^ It has also been hypothesized that improving muscle pathology could lead to preservation of proprioceptive synapses onto motor neurons that are lost in SMA.^95^ Therefore, muscle is considered a promising therapeutic target via numerous strategies: myostatin inhibition, activating fast troponin complexes, modulating metabolic and ergogenic pathways, and enhancing mitochondrial function (Figure 1).

Myostatin is a negative regulator of muscle growth, and inhibition of the myostatin signaling pathway has shown promising results, especially in less severe models of SMA^96^ or in addition to SMN-restoring therapies.^95^^,^^97^ Numerous inhibitory strategies have been trialed, such as antibodies directed against myostatin (or its precursors),^97^ against the myostatin receptor (activin type 2 receptor B [ActRIIB]),^96^ or using follistatin^98^ as an endogenous antagonist of myostatin. Because of high homology between myostatin and other growth factors of the transforming growth factor β (TGF-β) family, high specificity against myostatin is necessary to avoid previously observed side effects of the first clinically tested compounds in Duchenne muscular dystrophy (DMD) (gingival bleeding, telangiectasias, and hormonal level changes).^99^ A selective myostatin inhibitor (SRK-015) has shown promise in SMA mice^97^ and has been tested in type 2/3 SMA patients (NCT03921528; Table 2). Interim results revealed an increase in motor function in patients receiving the high dose, with no significant adverse effects.^100^ An ActRIIA/B ligand trap is also currently in phase I (BIIB110).

Fast skeletal muscle troponin activators prolong calcium binding to the troponin complex of fast skeletal muscle, hence increasing muscle contractility and reducing the energetic cost of contraction.^101^ This target is the basis of numerous small molecules being developed by Cytokinetics. Unfortunately, the results of a phase II study in SMA type 2–4 patients treated with reldesemtiv (CK-2127107) were not promising; of the 10 primary outcomes, only an improvement in maximum expiratory pressure was observed (NCT02644668).

Another approach is modulation of systemic anabolic pathways as an ergogenic strategy. For example, insulin growth factor-1 (IGF-1) has an anabolic effect on various tissues, including the CNS and muscle, and there is some evidence that enhancing IGF-1 signaling could be beneficial in SMA.^102^ However, a trial in SMA type 2/3 patients treated with synthetic somatotropin (growth hormone, leading to increased IGF-1 expression) did not show any improvement in muscle function.^103^ Similarly, the transcription factor KLF15, regulating metabolic and ergogenic muscular pathways, was downregulated in SMA mouse models, although the first preclinical trials only showed limited effect (Table 2).^104^

### Targeting the NMJ

SMA is associated with an impairment of NMJ development, maturation, and function, which contributes to muscle weakness and fatiguability.^76^ In this section, we focus on therapies specifically targeting the NMJ. Other therapeutic strategies can also improve NMJ pathology, e.g., by enhancing cytoskeleton dynamics (see Cytoskeleton section below and Figure 1).

The agrin/MuSK signaling pathway, which plays a key role in the formation and maturation of the NMJ, is dysregulated in SMA. Overexpression of agrin, or its downstream mediators such as DOK7, improves NMJ structure and reduces disease severity in SMA mice.^105^^,^^106^ Subcutaneous administration of NT-1654, the active portion of agrin, also delayed SMA mouse disease progression.^107^

Increased fatigability is a major symptom in SMA type 2/3 patients, and repeated nerve conduction studies show a decrement, confirming that NMJ dysfunction could be playing a key role in fatigability.^108^ Consequently, a phase II clinical trial^63^ is currently assessing the effects of pyridostigmine, an acetylcholinesterase inhibitor routinely prescribed for myasthenia gravis to slow degradation of acetylcholine within the synaptic cleft, increasing the cholinergic transmission efficiency. Preliminary reports suggested that pyridostigmine reduces fatigability in patients and should be considered as a possible adjunctive therapy (Table 2).^109^

Salbutamol, a β2-adrenoreceptor agonist, modestly improves muscle strength in patients by increasing levels of *SMN2* full-length mRNA and protein^110^ and possibly through stabilization of acetylcholine receptor clusters at the NMJ.^111^ Although small clinical studies suggest that it could help maintaining motor function^64^ or improve respiratory function^112^ of SMA type 2 patients, large-scale, placebo-controlled studies are lacking.

Improving neurotransmission at the NMJ is another possible therapeutic strategy. By blocking potassium channels, 4-aminopyridine prolongs the presynaptic action potential and increases acetylcholine release at the NMJ, thereby improving neurotransmission and motor function in a *Drosophila* model of SMA.^113^ However, a recent pilot study could not detect any improvement on locomotion in a group of ambulatory adult SMA patients.^114^ Amifampridine (3,4-DAP), another aminopyridine approved for the treatment of Lambert-Eaton myasthenic syndrome, has also been evaluated in ambulatory SMA type 3 patients (NCT03781479). Enrollment is complete, but results are yet to be released.

Finally, calcium signaling is also altered in nerve terminals of SMA mice and is associated with decreased neurotransmitter release.^115^ By slowing down the closure of the voltage-gated calcium channels, R-roscovitine increases presynaptic calcium influx.^116^ This resulted in increased survival and improvement of NMJ morphology in an SMA mouse model.

### Targeting the cytoskeleton

The cytoskeleton plays a key role in maintaining compartmentalization and polarization of neurons. In SMA, aberrant upregulation of the RhoA/Rho kinase (ROCK) pathway leads to disruption of actin pathways, affecting neuronal growth, differentiation, and regeneration.^117^ Pharmacological inhibition of the ROCK pathway by Y-27632^118^ and the FDA-approved drug fasudil^119^ improved survival, NMJ maturation, and muscle development in an intermediate mouse model of SMA. One of the downstream mediators of the ROCK pathway, PTEN, could be another potential target.^120^ Microtubule dynamics is also affected in SMA as stathmin-1, a microtubule-destabilizing protein, is a disease modifier and overexpression improved survival, motor function, and NMJ pathology in an SMA mouse model.^121^

### Targeting endocytosis

There is a growing body of evidence suggesting that perturbation of endocytosis plays an important role in SMA pathophysiology.^122^^,^^123^ Plastin 3 (PLS3), an actin-bundling protein,^124^ and neurocalcin delta (NCALD), a neuronal calcium sensor and negative regulator of endocytosis,^123^ have been identified as two strong SMN-independent protective modifiers in SMA patients. Overexpressing PLS3 partially rescued motor neuron pathology, especially NMJ structure and function, over a wide range of animal models of SMA,^122^^,^^124^ although it was insufficient to reverse the pathology in a severe mouse model.^125^ Similar therapeutic benefits were obtained by decreasing levels of NCALD.^126^ Furthermore, PLS3 interacts with coronin 1C—an actin-bundling protein, with calcineurin-like EF-hand protein 1 (CHP1)—a calcium sensor and calcineurin inhibitor,^127^ and with members of the hnRNP F/H family of proteins.^128^ Modulation of their respective expression improved impaired endocytic pathways and neuromuscular pathology in SMA models. Considering the overall consistent effect of their modulation on SMA animal models—especially the less severe ones—these represent a particularly attractive target for combinatorial therapy.

### Cell death mechanisms

#### Autophagy, ubiquitin homeostasis, and apoptosis

Core pathways in cell homeostasis, including autophagy, ubiquitin homeostasis, and apoptotic pathways, have been linked to neurodegeneration in SMA. Autophagy is a finely tuned system as either excessive or insufficient activity can be pathological. In SMA models, autophagosome numbers are increased.^129^^,^^130^ However, it remains unclear whether autophagic flux is increased or decreased.^129^^,^^131^ Further studies are warranted as autophagy could represent a therapeutic target. Indeed, administration of 3-methyladenine (an autophagic inhibitor) delayed motor neuron degeneration and subtly increased the lifespan in a severe mouse model.^130^ Additionally, calpains, a calcium-dependent family of proteases, regulate numerous cellular processes, including autophagy. Evidence suggests that calpains are overactivated in SMA and inhibition of calpain with calpeptin significantly increased survival and motor activity in SMA mouse models.^132^

The other key mechanism for protein degradation is the ubiquitin pathway. Marked dysregulation of this pathway has been shown in SMA, and mutations within one of the only two known E1 ubiquitin-activating enzymes, UBA1, is enough to induce X-linked SMA, a rare disorder with similar clinical symptoms to SMA, but not associated with *SMN* mutations.^133^ Disrupted ubiquitination has been highlighted as a key driver of SMA pathophysiology. A decrease in UBA1 activity is consistently observed across SMA models^134^^,^^135^ and restoration of UBA1 activity markedly ameliorated the phenotype of zebrafish and mouse models of SMA.^134^ This pathway therefore represents a powerful SMN-independent therapeutic target, but future research identifying small molecules that can stabilize or activate UBA1 will be required to facilitate development as an adjunctive therapy. Moreover, SMN itself is degraded via the ubiquitin system and pharmacological inhibition of SMN ubiquitination by ML372, an E3 inhibitor, increased SMN half-life and thus the lifespan of a severe SMA mouse model.^136^

The JNK signaling pathway plays a pivotal role in neuronal apoptosis and is a therapeutic target for multiple neurodegenerative disorders.^137^ The JNK pathway has been shown to be activated in spinal cord of SMA mice and patients,^138^ although a more recent study disputed these findings.^139^ Nevertheless, the fact that genetic^138^ and pharmacological inhibition^140^ of the JNK pathway resulted in improved lifespan and motor function would suggest that this may be a therapeutically relevant pathway in SMA.

#### p53 cell death pathway

Loss of SMN activates the tumor suppressor p53.^141^ The p53 pathway therefore presents a potentially attractive therapeutic target, since it is reported to drive motor neuron cell death in a severe mouse model of SMA.^142^ Loss of SMN may lead to downstream reduction of the endoplasmic reticulum (ER)-localized transmembrane protein Stasimon, thus activating p53.^141^ This same study showed that overexpression of Stasimon was sufficient to block motor neuron degeneration, as mice overexpressing Stasimon showed improved motor function and increased motor neuron numbers.^141^ However, the extent to which this rescue is truly SMN independent is unclear, since further analysis of the subset of mice that showed improved motor function also found twice the levels of full-length *SMN2* RNA transcripts compared with the non-responding group.^141^ Decreased Stasimon expression in SMA models is thought to be due to loss of SMN-mediated U12 intron splicing, and delivery of minor small nuclear RNA (snRNA) genes to boost this splicing pathway improved survival and motor function in SMA mice.^143^ This pathway could also be targeted therapeutically via inhibition of p38 using MW150, thereby preventing phosphorylation and activation of p53.^141^ The p38 pathway is also the proposed mechanism of action for celecoxib, an NSAID shown to extend the lifespan of an intermediate mouse model^144^ that reached phase II clinical trials, but was recently prematurely terminated with results yet to be published (NCT02876094).

### Other potential therapeutic targets

#### Neurite outgrowth

The transmembrane protein chondrolectin (Chodl), involved in axonal guidance, neurite outgrowth, and synaptogenesis, is dysregulated in SMA.^145^^,^^146^ Overexpression of Chodl rescued motor neuron pathology in a zebrafish model.^145^ However, further experiments, especially in mammalian models of SMA, are required to confirm the therapeutic potential of this approach.

The cyclin-dependent kinase 5 (CDK5), involved in neuronal architecture maintenance, neurite outgrowth, and synaptic plasticity, is overactivated over a wide range of neurodegenerative disorders, including SMA. Therefore, pharmacological inhibitors of CDK5 could be particularly attractive.^147^ In SMA and other neurodegenerative disorders, CDK5 hyperactivity leads to tau hyperphosphorylation. The fact that knocking out tau could ameliorate motor neuron degeneration and synaptic stripping in an SMA mouse model further emphasizes the potential role played by CDK5 and tau in SMA pathophysiology.^148^

#### miRNAs

Micro-RNAs (miRNAs) are regulatory RNA molecules with diverse and interacting roles in the regulation of the cell’s internal environment and can be either blocked or overexpressed to drive a particular pathway. These strategies are being explored across neurodegenerative diseases. As one example, miR-206 drives regenerative pathways at the NMJ in motor neuron disorders and is upregulated at late stages of disease in SMA mouse models as a pro-survival mechanism, but not sufficiently to rescue motor neuron degeneration.^149^ Overexpression of miR-206 extended the lifespan of SMA mice and improved motor performance, suggesting a possible therapeutic option.^150^ miR-23a is another potential therapeutic target, since it was found to be downregulated in SMA iPSC-derived motor neurons and overexpression increased the lifespan of SMA mice.^151^

#### Lifestyle changes

Metabolic dysregulation is common across SMA mouse models and patient groups, incorporating dysregulation of lipids, amino acids, and glucose.^152^ As such, modulation of diet and/or exercise may be of significant therapeutic benefit. In a mouse model of mild SMA, both high-intensity swimming and low-intensity running showed benefits in terms of both lipid and glucose metabolism.^153^ The therapeutic benefits of exercise would appear be most applicable to older SMA patients with less severe forms of the disease, or patients with extended survival resulting from treatment with SMN replacement therapies. In particular, lifestyle changes offer a relatively easy (and cheap) way to deliver SMN+ combinatorial therapies.

## Future perspectives

Over the past few years, the field of SMA research has been revolutionized. Thanks to the development of ground-breaking SMN replacement strategies, there are finally good therapeutic options for families with children diagnosed with SMA, albeit at an extremely high cost.^8^ These therapies are completely changing the phenotype of the treated patients, who will no longer follow the natural history of SMA. With a prolonged lifespan and improved neuromuscular function, non-CNS symptoms could become more of a concern in those treated with CNS-targeting therapies.^6^ However, regardless of the pioneering nature of these therapies, it is becoming obvious with hindsight and long-term follow-up of the first treated patients that SMN protein replacement is not a cure. The timing of treatment is critical, with early treatment having much better prospects,^154^ but there also appears to be a subsection of patients who do not respond.^23^ Several factors may contribute to this variable response, including genetic factors beyond *SMN1* and *SMN2*, environmental factors, or access to and quality of medical care. Some patients have already resorted to combining the various available SMN replacement therapies, with no evidence yet for additional benefit, although clinical trials are ongoing and recruiting at the time of writing.

It is now well acknowledged that to improve the chances of a good response to SMN replacement therapy, treatment must be initiated as early in life as possible. The gold standard for SMA treatment should therefore involve neonatal genetic screening, as currently practiced in limited countries in the world. There has even been recent evidence for developmental pathology *in utero* in SMA mouse models,^155^ further highlighting that early treatment is key. The SMA field has been captivated over recent years with the development of these SMN replacement therapies. After a few years of clinical experience, we have seen how life changing these drugs can be, but we are also aware of their limitations, especially for those patients diagnosed later in life or suffering from a milder form of SMA. This calls for SMN+ strategies that include SMN-independent therapies, such as those described in this review. Several of these targets, due to their broadly neuroprotective actions, could also be of benefit to other neurodegenerative disorders, particularly other motor neuron diseases. This cross-disease approach, particularly focusing on repurposing drugs with known safety profiles (Table 2), could drive therapies faster along the path to the patient.

Because of the ubiquitous roles of SMN in the cell,^5^ it is not surprising that numerous therapeutic targets are being identified. Animal experiments have shown that a lot of these targets may have more efficacy in milder forms of the disease. This not only advocates for their use as a combinatorial therapy but also calls for a reassessment of previous targets whose effects may have been overlooked when they could not overcome the particularly severe phenotype alone. Currently, the therapeutic potential of most of these targets has been evaluated by genetic manipulation in animal models, and future emphasis should be placed on bridging the gap between target discovery and small-molecule development.

## Conclusions

The three currently approved drugs for SMA replacement therapy have given life-changing treatment options to SMA patients and their families for the first time. All three treatments extend life expectancy and allow patients to reach motor milestones that would previously have been unachievable. However, the limitations of these therapies are now apparent, opening the road for development of wider targets beyond SMN replacement. Before the efficacy of gene replacement and/or splice modulation was confirmed in clinical trials, alternative SMN-dependent and SMN-independent strategies were investigated. These may still play an important role in SMA therapy, allowing both combinatorial and systemic approaches to be developed. Such approaches targeting pathophysiological events occurring in SMA may also have benefits for other neurodegenerative and neuromuscular diseases. The development of SMN replacement therapies is not the end of the road for SMA therapy development. On the contrary, they have opened a new world of possibilities.
